# Complications of tunneled and non-tunneled peripherally inserted central catheter placement in chemotherapy-treated cancer patients: a meta-analysis

**DOI:** 10.3389/fsurg.2024.1469847

**Published:** 2024-10-15

**Authors:** Jiana Hong, Xiaodan Mao

**Affiliations:** Department of Medical Oncology, The First Affiliated Hospital of Zhejiang Chinese Medical University (Zhejiang Provincial Hospital of Traditional Chinese Medicine), Hangzhou, Zhejiang, China

**Keywords:** PICC, cancer, complications, tunneled, meta-analysis

## Abstract

**Background:**

Tunneled peripherally inserted central catheters (PICC) have potential to reduce complications compared to non-tunneled PICC in previous studies. Which is better is debatable. Thus, the aim to compare the effect of tunneled and non-tunneled PICC for cancer patients undergoing chemotherapy.

**Methods:**

Embase, PubMed, Cochrane Library database, and CNKI were searched from inception to March 15, 2024. Odds ratios (ORs) with 95% confidence intervals (95% CIs) was calculated to assess the complications of tunneled and non-tunneled PICC for cancer patients undergoing chemotherapy using random- or fixed-effects models.

**Results:**

A total of 12 articles were retrieved. Meta-analysis showed that tunneled PICC significantly decreased the risk of wound oozing (OR: 0.29, 95% CI: 0.20–0.41), infection risk (OR: 0.41, 95% CI: 0.20–0.85), thrombosis risk (OR: 0.26, 95% CI: 0.15–0.44), phlebitis risk (OR: 0.23, 95% CI: 0.13–0.40), and catheter dislodgement risk (OR: 0.33, 95% CI: 0.22–0.50) compared to non-tunneled PICC.

**Conclusions:**

The subcutaneous tunneling technology has advantages over normal technique in decreasing PICC-related complications for cancer patients undergoing chemotherapy.

**Systematic Review Registration:**

PROSPERO (CRD42024522862).

## Introduction

Cancer has become the second leading cause of death worldwide, posing a serious threat to people's lives and health, and its incidence is increasing annually (1). Chemotherapy is the standard treatment for cancer patients and can prolong their survival (2). However, long-term chemotherapy causes significant damage to the body of cancer patients (3). Prolonged intravenous infusion can cause vascular injury and increase the risk of catheter-related bloodstream infections (4, 5). Currently, traditional peripherally inserted central catheter (PICC) techniques are widely used in the chemotherapeutic treatment of cancer patients (6, 7). Traditional PICC methods have advantages, but the issue of catheter-related infections cannot be ignored because they can lead to infective endocarditis, septic embolism, and even death (8, 9). Another infection complication that causes 30% of traditional PICC treatment failures is catheter-related thrombosis (CRT), which is associated with hypercoagulability and endothelial vascular injury (10). Furthermore, if there are poor vascular conditions in the mid-upper arm or scarred skin in the puncture area, traditional non-tunneled PICC may result in changes in the puncture area, ultimately leading to puncture near the axilla (11). However, puncture in this area increases the probabilities of catheter displacement, dislodgement, and bloodstream infections.

In recent years, subcutaneous tunneling techniques have attracted a considerable amount of attention. In tunneled PICC, the upper 1/3 of the arm is the puncture site, and the external part of the catheter is passed through a subcutaneous tunnel to the middle 1/3 of the arm, thus achieving the optimal exit position for the catheter (12, 13). This is because the middle arm provides the greatest stability, thereby lowering the risks of infection, venous thrombosis, catheter displacement, and other complications associated with conventional PICC placement (14). However, only a few studies have compared the outcomes of tunneled and non-tunneled PICC during adjuvant chemotherapy (15–17). Currently, there is no clear or consistent evidence suggesting which treatment is safer or preferable. Therefore, the aim of this meta-analysis was to compare the outcomes of tunneled and non-tunneled PICC placement during cancer chemotherapy treatment and provide useful information for physicians to better counsel cancer patients.

## Materials and methods

This meta-analysis was conducted according to the Preferred Reporting Items for Systematic Reviews and Meta-Analyses (PRISMA) statements (18). Ethical approval is not required due to all the data analysis based on the published data. This meta-analysis was registered in the International prospective register of systematic reviews (PROSPERO registration number: CRD42024522862).

### Literature search

The literature search was performed using the Embase, PubMed, Cochrane Library database, and CNKI from inception to March 15, 2024. We used Boolean logic with keywords or MeSH terms included PICC, peripherally inserted central catheter, tunneled, cancer, and chemotherapy. References of the included studies were checked for additional potentials studies.

### Study selection

The inclusion criteria were as follows: (1) population: chemotherapy-treated cancer patients; (2) intervention: tunneled PICC; (3) comparison: non-tunneled PICC; (4) outcome: wound oozing, thrombosis, infection, phlebitis, catheter dislodgement, and catheter occlusion; (5) study design: randomized controlled trial (RCT). The exclusion criteria were as follows: (1) incompletely reported data; (2) duplicate previous literature; (3) conference abstracts, comments, or reviews.

### Data extraction

Two reviewers independently extracted information from included studies using a standardized electronic form. Any disagreements were resolved through discussion with a third reviewer. The following information was extracted: first author, study design, groups, gender, age, sample size, and outcome.

### Quality assessment

Two reviewers independently assessed the risk of bias of the included RCTs using the Cochrane Collaboration risk of bias tool (19). It contains six perspectives including random sequence generation, allocation concealment, blinding of participants and personnel, blinding of outcome assessment, selective reporting, and other bias risk. Each perspective was judged as “low”, “high”, or “unclear” risk.

### Statistical analysis

Statistical analyses were undertaken using Stata software version 12.0 (Cochrane Collaboration, Oxford, UK). The random- or fixed effect model was used to calculate the odds ratio (OR) with a 95% confidence interval (95% CI). The *I*^2^ and chi-square tests were used to assess the heterogeneity of the studies. The *I*^2^ < 25%, 25% ≤ *I*^2^ < 50%, 50% ≤ *I*^2^ < 75%, and *I*^2^ ≥ 75% indicated no heterogeneity, low heterogeneity, moderate heterogeneity, and high heterogeneity, respectively. If heterogeneity is observed, the random-effects model is used, otherwise, a fixed effect model was selected for analysis. The publication biases were judged by Egger test and Begg test. Sensitivity tests were also conducted to examine the robustness of the disparities.

## Results

### Study selection

**Figure 1:**
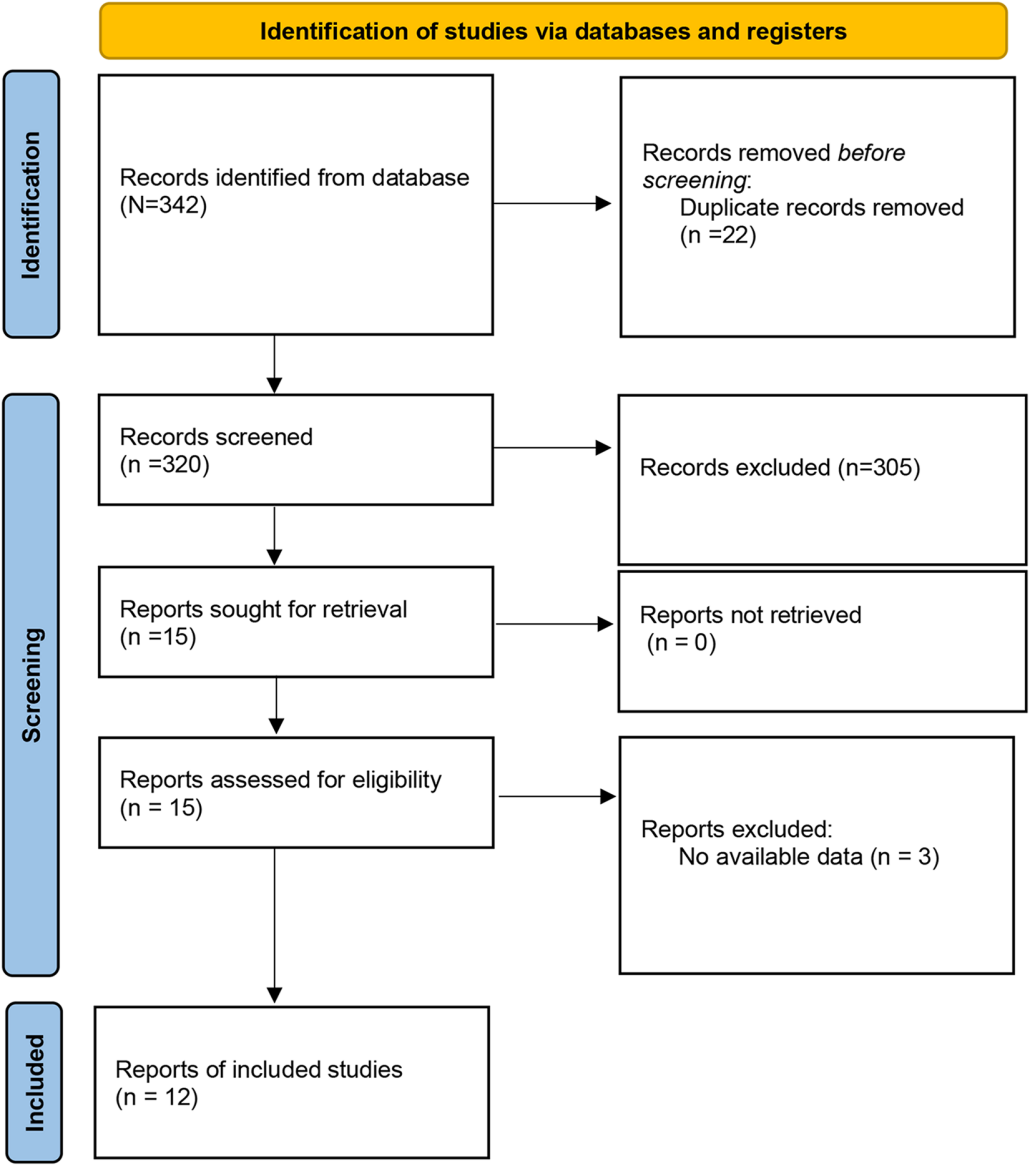
Selection process of included studies.

The literature search yielded 342 articles. After removing 22 duplicated studies, 320 articles that potentially investigated tunneled and non-tunneled PICC in chemotherapy-treated cancer patients were screened. After screening the title and abstract, 305 articles were removed, and 15 studies were eligible for full-text review. Of these, 3 studies were excluded. Finally, 12 RCTs (16, 17, 20–27) with 2,940 participants (tunneled PICC 1,484 vs. non-tunneled PICC 1,456) were included in this meta-analysis (Figure 1).

### Study characteristics

The characteristics of the included studies were presented in Table 1. These studies were published between the years of 2017 and 2024. Eleven studies were conducted in the China and 1 in the Greece. The sample sizes of the trials ranged from 30 to 493. The mean age of the participants ranged from 37.57 to 68.57 in the tunneled PICC group and 40.96 to 64.17 in the non-tunneled PICC group. The 1,377 participants in the tunneled PICC group and 1,359 in the non-tunneled PICC group. The outcomes index contains wound oozing, thrombosis, infection, phlebitis, catheter dislodgement, and catheter occlusion.

**Table 1:** Characteristics of the included studies.

Author, year	Study design	Groups	Gender (male/female)	Age (years)	Sample size	Outcome
Xiao et al. 2021 (20)	RCT	Tunneled PICC	35/19	45.64 ± 11.59	64	Wound oozing, thrombosis, infection, phlebitis, catheter dislodgement, catheter occlusion
		Non-tunneled PICC	39/26	47.95 ± 11.96	65	
Dai et al. 2020 (21)	RCT	Tunneled PICC	51/36	45.70 ± 11.32	87	Wound oozing, thrombosis, infection, phlebitis, catheter dislodgement, catheter occlusion
		Non-tunneled PICC	55/32	45.66 ± 11.45	87	
Sheng et al. 2024 (16)	RCT	Tunneled PICC	60/278	55.37 ± 12.48	338	Wound oozing, thrombosis, infection, phlebitis, catheter dislodgement
		Non-tunneled PICC	53/285	54.84 ± 13.90	338	
Maria et al. 2019 (17)	RCT	Tunneled PICC	14/16	55.17 ± 9.36	30	Thrombosis, infection
		Non-tunneled PICC	17/13	54.47 ± 9.18	30	
Meng et al. 2017 (26)	RCT	Tunneled PICC	120/108	49.2 ± 6.8	228	Infection, phlebitis, catheter dislodgement
		Non-tunneled PICC	113/109	48.8 ± 4.7	222	
Gao et al. 2023 (23)	RCT	Tunneled PICC	26/14	53.41 ± 7.53	40	Wound oozing, infection, phlebitis, catheter dislodgement
		Non-tunneled PICC	22/18	52.67 ± 7.29	40	
Huang 2023 (24)	RCT	Tunneled PICC	19/20	37.57 ± 5.67	39	Infection, phlebitis, catheter dislodgement, catheter occlusion
		Non-tunneled PICC	20/19	40.96 ± 5.23	39	
Fan et al. 2020 (22)	RCT	Tunneled PICC	21/17	51.42 ± 2.66	38	Wound oozing, phlebitis, catheter dislodgement
		Non-tunneled PICC	20/18	51.34 ± 2.71	38	
Li 2023 (25)	RCT	Tunneled PICC	15/15	50.56 ± 8.96	30	Wound oozing, thrombosis, infection, phlebitis
		Non-tunneled PICC	17/13	51.49 ± 8.11	30	
Wang et al. 2019 (27)	RCT	Tunneled PICC	257/236	55.92 ± 11.77	493	Wound oozing, thrombosis, infection, catheter dislodgement, catheter occlusion
		Non-tunneled PICC	255/215	55.84 ± 11.41	470	
Peng 2024 (35)	RCT	Tunneled PICC	30/27	68.57 ± 6.05	57	Wound oozing, thrombosis, phlebitis
		Non-tunneled PICC	33/24	64.17 ± 7.23	57	
Wang et al. 2024 (36)	RCT	Tunneled PICC	26/14	54.19 ± 4.50	40	Wound oozing, thrombosis, infection, phlebitis, catheter dislodgement
		Non-tunneled PICC	25/15	55.58 ± 3.47	40	

RCT, randomized controlled trials; PICC, peripherally inserted central catheter.

### Results of quality assessment

**Figure 2:**
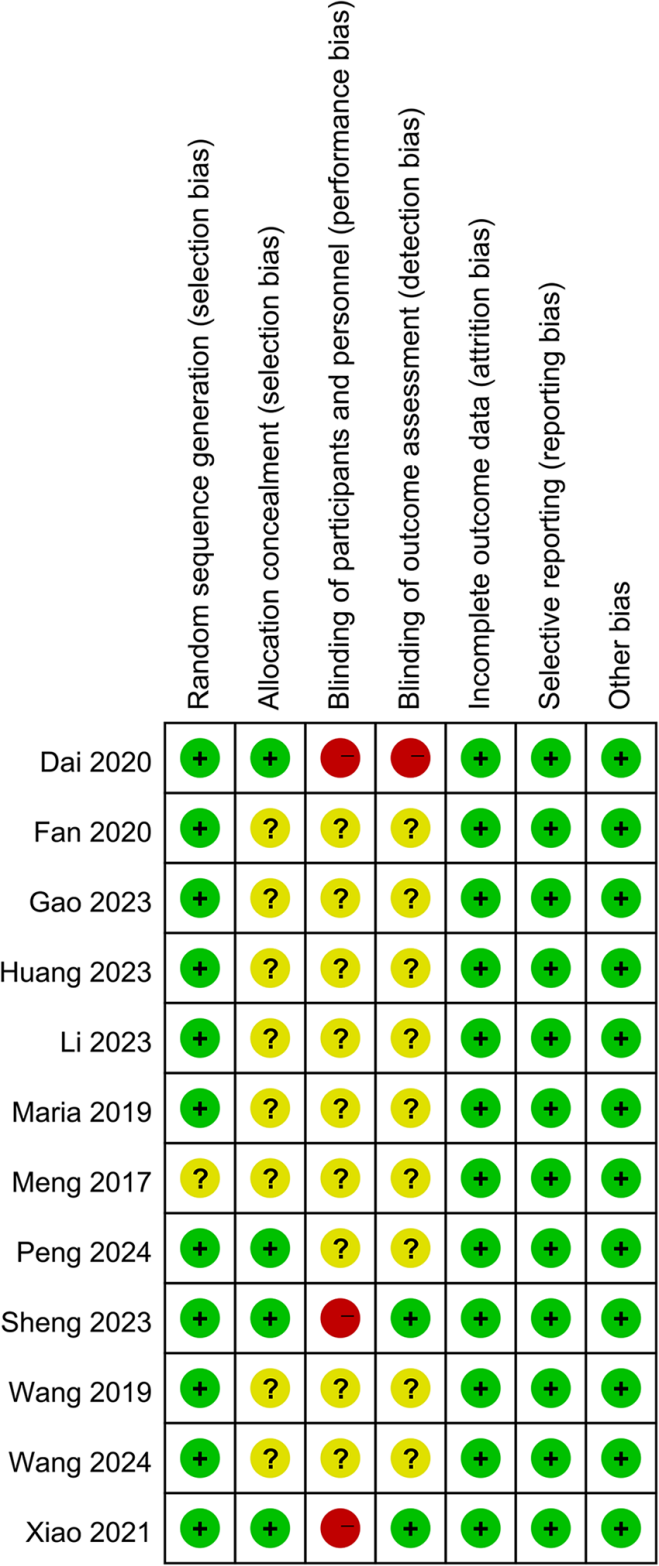
Summary of risk of bias for each included study.

The Cochrane risk of bias assessment tool was used to evaluate risk in the included studies. Eight studies didn't describe allocation concealment, blinding of participants and personnel, and blinding of outcome assessment. One study didn't report random sequence generation. Two studies had blinded outcome assessments and none blinded participants or personnel (Figure 2).

### Meta-analysis results

**Figure 3:**
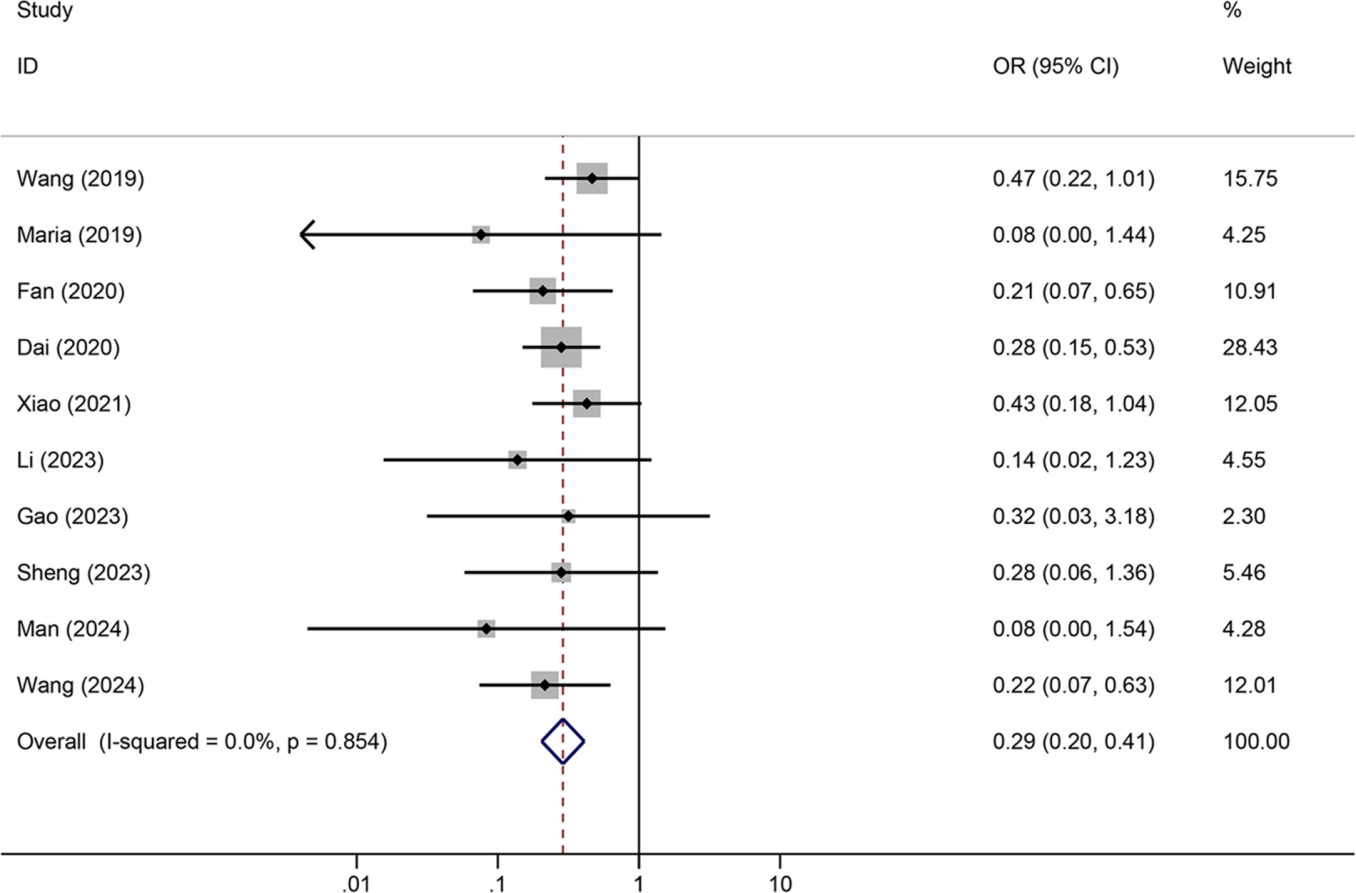
Forest plots of the impact of tunneled PICC on wound oozing.

**Figure 4:**
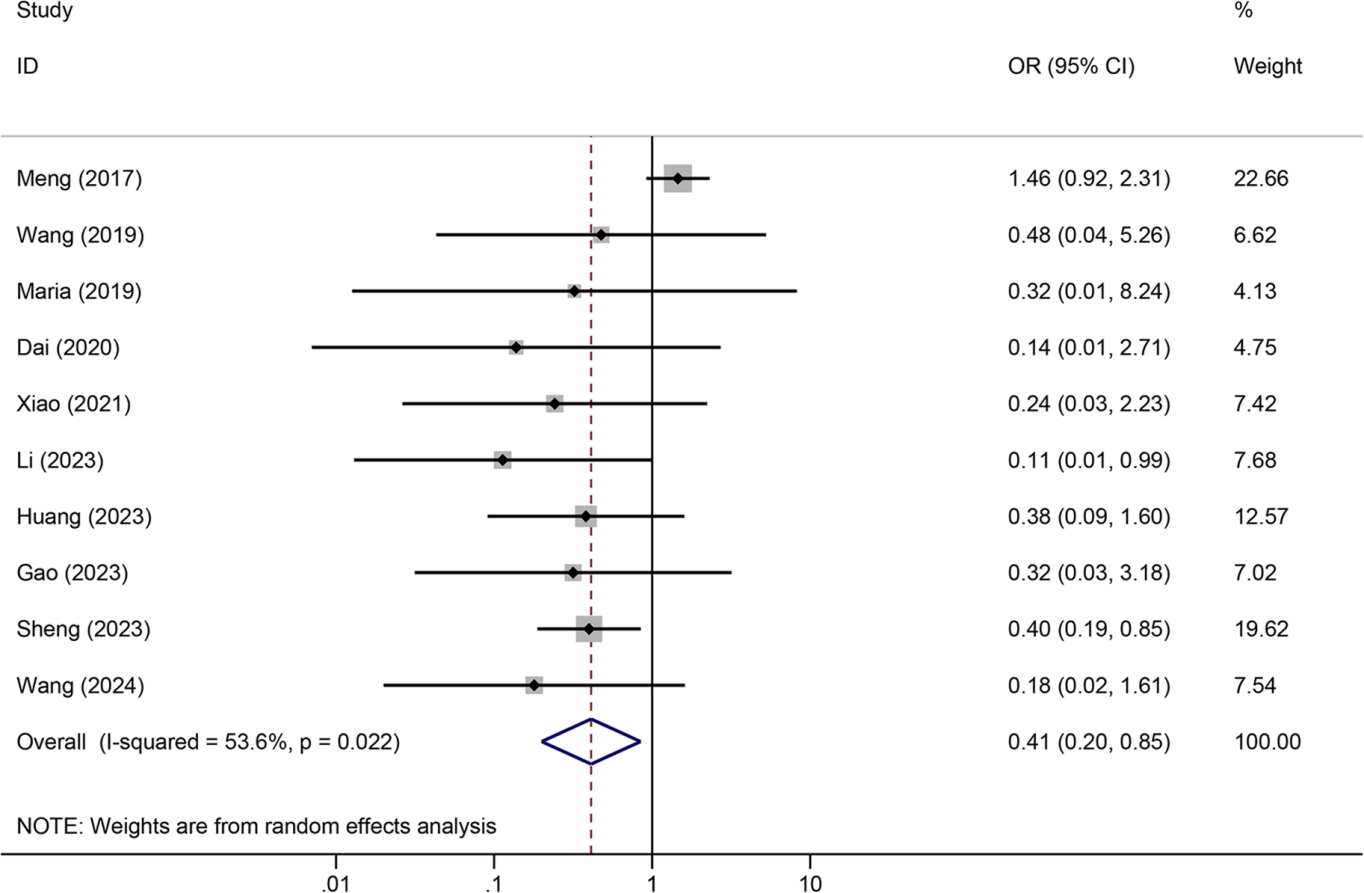
Forest plots of the impact of tunneled PICC on infection.

**Figure 5:**
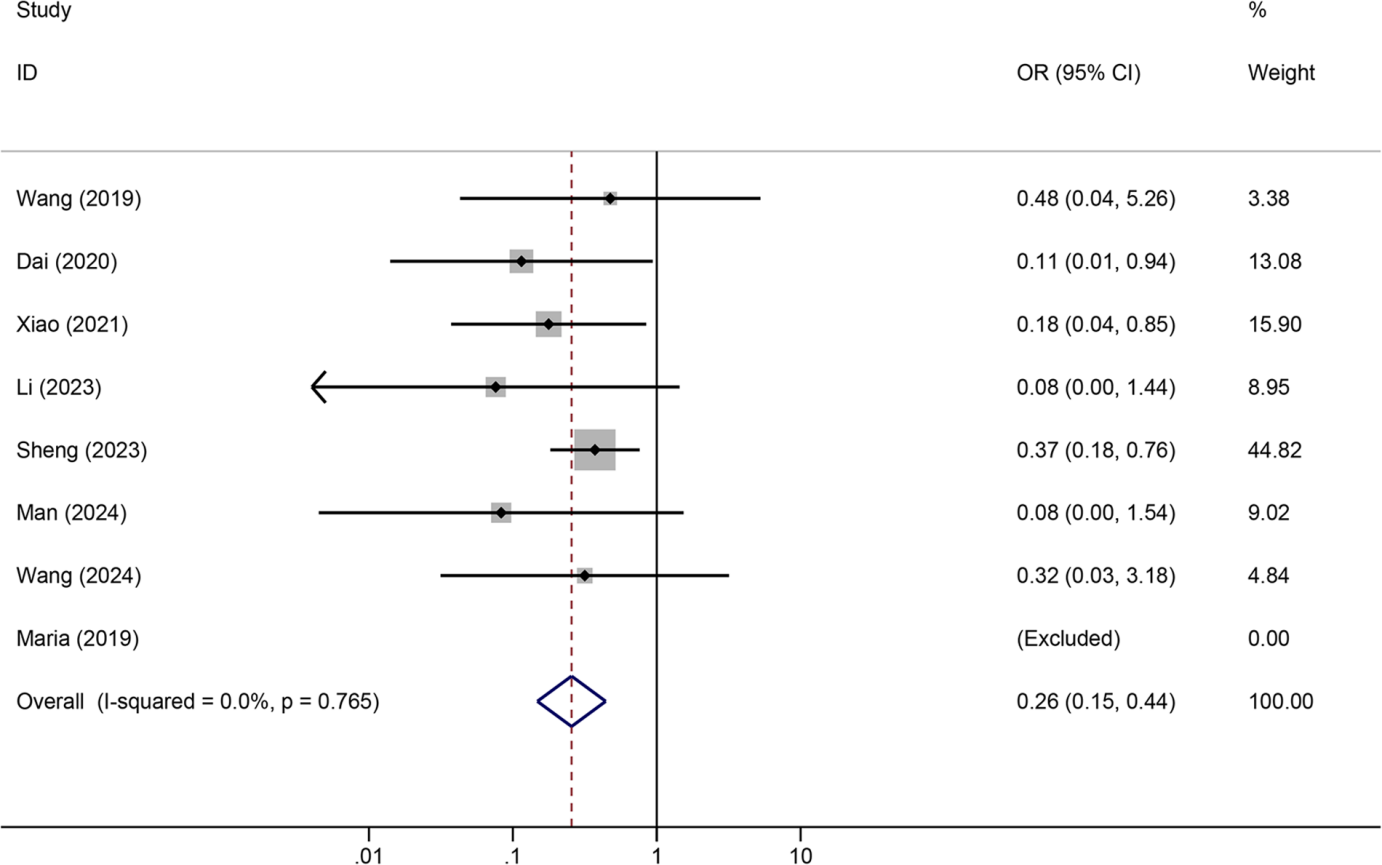
Forest plots of the impact of tunneled PICC on thrombosis.

**Figure 6:**
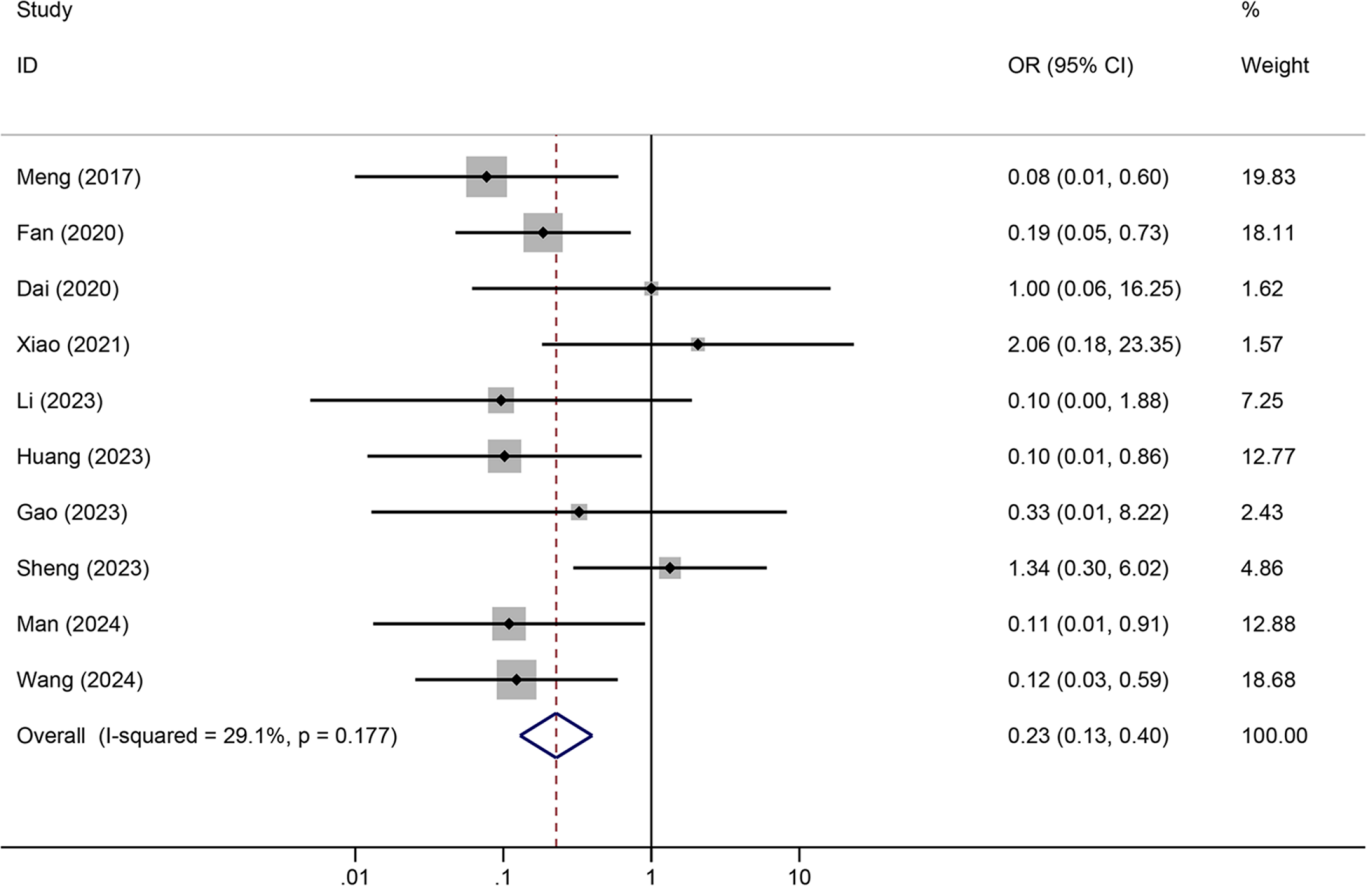
Forest plots of the impact of tunneled PICC on phlebitis.

**Figure 7:**
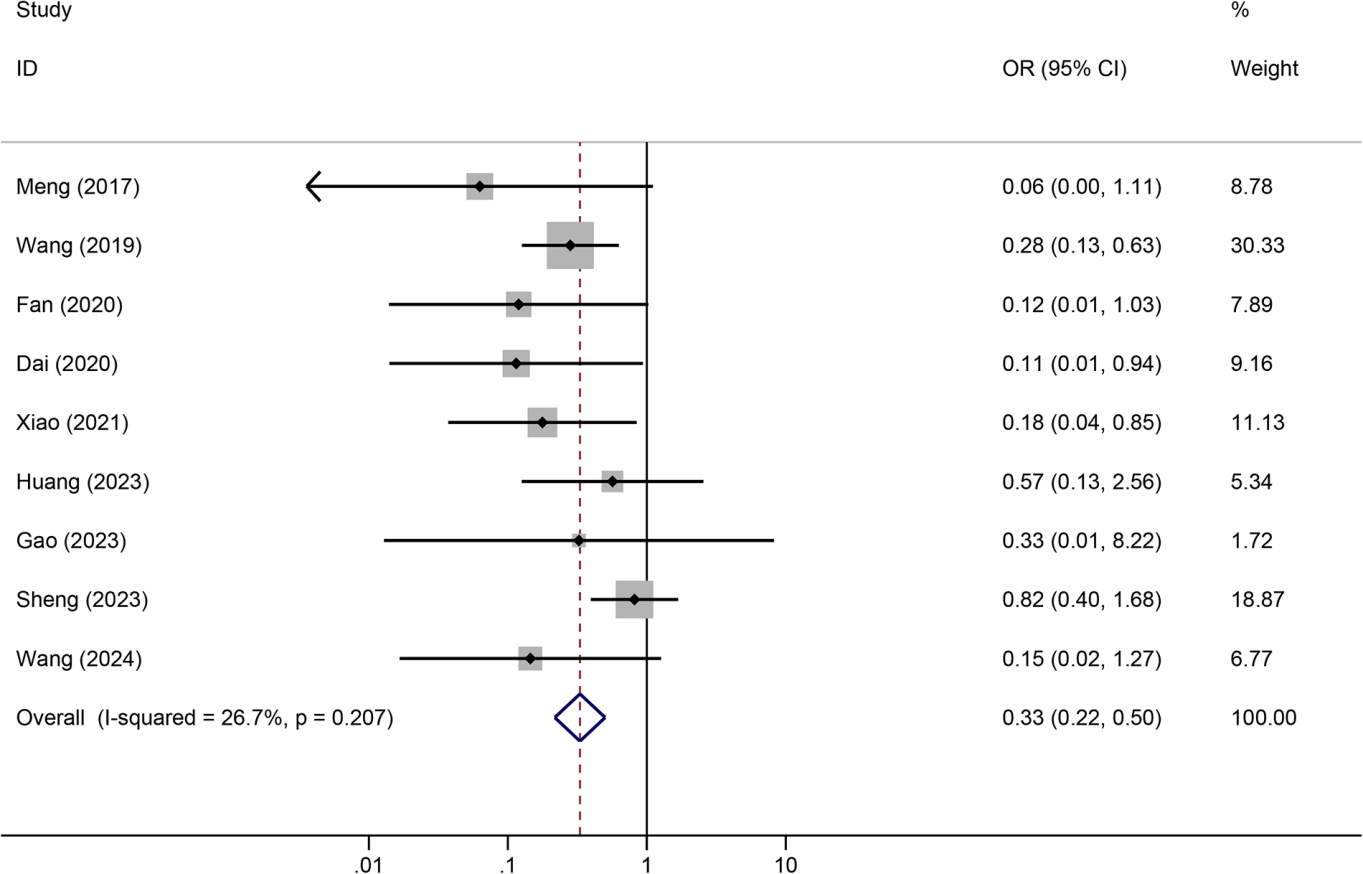
Forest plots of the impact of tunneled PICC on catheter dislodgement.

Tunneled PICC significantly decreased the risk of wound oozing (OR: 0.29, 95% CI: 0.20–0.41, *p* < 0.001) with low heterogeneity (*I*^2^ = 0%), infection risk (OR: 0.41, 95% CI: 0.20–0.85, *p* = 0.032) with moderate heterogeneity (*I*^2^ = 54.6%), thrombosis risk (OR: 0.26, 95% CI: 0.15–0.44, *p* < 0.001) with low heterogeneity (*I*^2^ = 0%), phlebitis risk (OR: 0.23, 95% CI: 0.13–0.40, *p* < 0.001) with moderate heterogeneity (*I*^2^ = 29.1%), and catheter dislodgement risk (OR: 0.33, 95% CI: 0.22–0.50, *p* < 0.001) with moderate heterogeneity (*I*^2^ = 26.7%) compared to non-tunneled PICC (Figures 3–7). However, no significant difference was observed in catheter occlusion risk (OR: 0.82, 95% CI: 0.49–1.37, *p* = 0.450) with moderate heterogeneity (*I*^2^ = 47.6%).

### Sensitivity analyses

**Figure 8:**
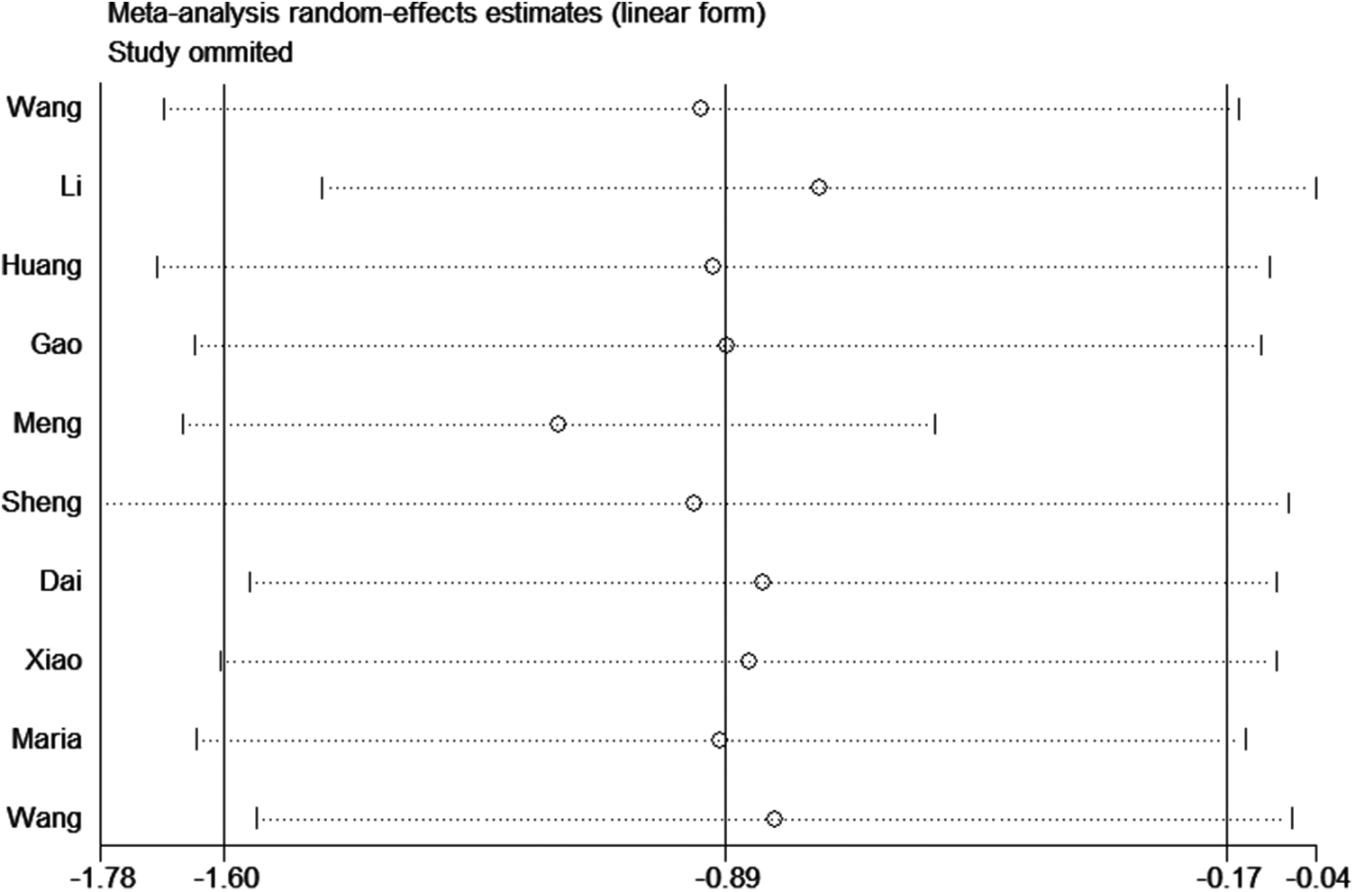
Sensitivity analysis of the impact of tunneled PICC on infection.

To assess the robustness of the results, we performed a sensitivity analysis by removing Meng et al. 2017. The heterogeneity significantly decreased (*I*^2^ = 0%) and the result was reliable (Figure 8).

### Publication bias

To assess the presence of publication bias in this meta-analysis regarding infection risk, visual funnel plots and Egger's regression test were employed. The funnel plots exhibited a balanced and symmetrical shape, suggesting no substantial publication bias. The statistical analysis further confirmed this finding, with the calculated *P*-value of the Egger test being 0.325, endorsing the absence of significant publication bias in the study.

## Discussion

Despite being an important decision in clinical practice, very little is known about the benefits and relative risks of various venous access methods for cancer patients receiving chemotherapy. This meta-analysis included 2,940 cancer patients receiving chemotherapy. Among these patients, 1,484 underwent tunneled PICC placement and 1,456 underwent non-tunneled PICC placement. Compared to non-tunneled PICC placement, tunneled PICC placement significantly reduced the incidences of wound leakage, infection risk, thrombosis, venous inflammation, and catheter dislocation.

In this study, the rates of wound leakage, infection, and catheter dislodgement were significantly lower in patients treated with the subcutaneous tunnel technique than in patients treated with the conventional method for placing PICCs, which is consistent with the findings of previous research (20). However, we found that the prevalence of catheter occlusion was not significantly different between tunneled PICC and non-tunneled PICC (OR: 0.82, 95% CI: 0.49–1.37, *p* = 0.450) in all subjects. Interestingly, although the study by Meng et al. (26) found that 7.4% of patients with tunneled PICC developed catheter occlusion compared with 6.7% of patients in the non-tunneled PICC group, other studies comparing tunneled PICC with non-tunneled PICC reported a decreased risk of PICC-associated catheter occlusion (24, 27). Part of the difference observed in rates of catheter occlusion among studies may be insufficient sample sizes and different tunnel lengths. Catheter dislodgement can be caused by coughing, vomiting, improper arm positioning, or skin issues. The presence of a subcutaneous tunnel increases the strength of the fixation of the catheter to the surrounding skin, reducing the mobility of the catheter and therefore reducing the dislodgement rate (28). Additionally, the tunnel that forms between the venous puncture site and the catheter exit site acts as a buffer and provides compression hemostasis, reducing the rate of bleeding. Furthermore, the subcutaneous tunnel increases the difficulty for microorganisms to travel retrograde along the catheter, reducing the incidence of infection (16). Moreover, the lower rate of catheter malpositioning reduces the introduction of pathogens due to repeated inward displacement of the catheter, and the lower rate of bleeding reduces the colonization of bacteria on the catheter, further controlling the occurrence of infection (29).

The ratio of catheter-to-vein diameter is an important factor affecting the occurrence of PICC-related venous thrombosis. The ratio of catheter to vein should be between 33% and 45% to reduce the incidence of thrombosis (30). The subcutaneous tunnel allows higher positioning of the puncture point, where the vein diameter is larger, and preserves an exit site in one-third of the arm for proper fixation (31). A larger vessel diameter not only helps reduce the incidence of thrombosis but also allows the use of multilumen catheters that would otherwise exceed the optimal ratio of catheter to vein for suitable vessels. A larger lumen diameter at the tunneled PICC puncture site reduces the incidence of thrombosis caused by repeated mechanical friction against the vessel wall and reduced blood flow (32). Additionally, vein wall damage is also a major cause of venous inflammation, and a lower catheter-to-vein diameter ratio reduces mechanical friction between the catheter and vessel wall, thereby reducing the occurrence of venous inflammation (12, 33).

Tunnel length is associated with the risk of PICC-related complications, and longer tunnel lengths increase the stability of tunnel passages (34). Earlier studies have found that PICC with a tunnel length of 5 cm significantly reduced the incidence of complications (20). However, PICC with a tunnel length of 6 cm did not show a greater advantage against in reducing bleeding event (15). In our included literatures, the length of the tunnel was between 3 and 5 cm. Recent study has compared different tunnel length for a tunneled PICC to reduce the risk of PICC-related complications and found that a longer tunnel length was associated with longer catheter residence times and fewer PICC-related complications, and a tunnel length longer than 4 cm was recommended for tunneled PICC (15).

Several limitations of this study should be noted. First, due to the limited number of studies, we were unable to determine whether the results were influenced by age or race. Second, there were insufficient data to compare the impact of different tunnel lengths on outcome measures; the nutritional status of the respondents as well as age, sex, and sex characteristics are potential sources of bias. Furthermore, our study did not evaluate the cost-effectiveness of these two regimens. Cost-effectiveness should also be considered when selecting tunnel PICC placement for cancer patients.

## Conclusions

In conclusion, for cancer patients undergoing chemotherapy, subcutaneous tunneling technology are a safer treatment option than non-tunneled PICC. However, due to limitations in the number and quality of the included research, the conclusions of this study need to be confirmed by using larger sample sizes, multicenter and high-quality clinical trials.

## Data Availability

The original contributions presented in the study are included in the article/Supplementary Material, further inquiries can be directed to the corresponding author.
